# DROPA: DRIP-seq optimized peak annotator

**DOI:** 10.1186/s12859-019-3009-9

**Published:** 2019-08-06

**Authors:** Marco Russo, Bruno De Lucca, Tiziano Flati, Silvia Gioiosa, Giovanni Chillemi, Giovanni Capranico

**Affiliations:** 1 Department of Pharmacy and Biotechnology, University of Bologna, Bologna, Italy; 2 National Council of Research, CNR, Institute of Biomembranes, Bioenergetics and Molecular Biotechnologies, Bari, Italy; 3 SCAI-Super Computing Applications and Innovation Department, CINECA, Rome, Italy; 4 Department for Innovation in Biological, Agro-food and Forest systems (DIBAF), University of Tuscia, Viterbo, Italy

**Keywords:** R-loop, Non-canonical DNA structures, Genome annotation, Next-generation sequencing

## Abstract

**Background:**

R-loops are three-stranded nucleic acid structures that usually form during transcription and that may lead to gene regulation or genome instability. DRIP (DNA:RNA Immunoprecipitation)-seq techniques are widely used to map R-loops genome-wide providing insights into R-loop biology. However, annotation of DRIP-seq peaks to genes can be a tricky step, due to the lack of strand information when using the common basic DRIP technique.

**Results:**

Here, we introduce DRIP-seq Optimized Peak Annotator (DROPA), a new tool for gene annotation of R-loop peaks based on gene expression information. DROPA allows a full customization of annotation options, ranging from the choice of reference datasets to gene feature definitions. DROPA allows to assign R-loop peaks to the DNA template strand in gene body with a false positive rate of less than 7%. A comparison of DROPA performance with three widely used annotation tools show that it identifies less false positive annotations than the others.

**Conclusions:**

DROPA is a fully customizable peak-annotation tool optimized for co-transcriptional DRIP-seq peaks, which allows a finest gene annotation based on gene expression information. Its output can easily be integrated into pipelines to perform downstream analyses, while useful and informative summary plots and statistical enrichment tests can be produced.

**Electronic supplementary material:**

The online version of this article (10.1186/s12859-019-3009-9) contains supplementary material, which is available to authorized users.

## Background

R-loops are three stranded nucleic acid structures composed by a DNA:RNA hybrid duplex and a displaced ssDNA (single strand DNA) strand. R-loops form co-transcriptionally when nascent RNAs anneal back to DNA template strand [1, 2]. R-loops have been shown to be involved in many nuclear processes such as transcription regulation, DNA methylation modulation and DNA repair mechanisms. However unscheduled R-loop formation is associated with DNA damage accumulation, genome instability and genetic diseases [3].

Genome-wide maps of these peculiar nucleic acid structures have boosted our understanding of R-loop biology [1]. Immunoprecipitation-based techniques, generally known as DRIP (DNA:RNA Immunoprecipitation), coupled with parallel sequencing (DRIP-seq), are widely used to maps R-loops genome-wide [4, 5] and several DRIP variants have been developed with the intent to improve the identification of genomic R-loops [3]. However, the most common technique (DRIP) allows the detection of R-loop regions without providing the strand information of the DNA:RNA hybrid. Understanding the DNA strand forming the hybrid is essential to investigate the dynamic interplay of R-loops with other nucleic acid structure (e.g. G-quadruplexes) [6] or with basic directional mechanisms such as replication and transcription [7].

Moreover, DRIP-seq data are commonly analyzed with standard peak callers, such as MACS (Model-based Analysis of ChIP-Seq) [8], to identify regions with above-threshold coverage signals, usually called “peaks”. Nevertheless, DRIP peaks are markedly different from traditional ChIP (Chromatin Immunoprecipitation) peaks of transcription factors as the former peaks are usually much longer than the latter ones, spanning across several genes features or different genes. As R-loops can span several gene features (REFs), the assignment of R-loop peaks to a unique feature may not be appropriate.

To overcome these issues, we have developed a new software, named DROPA (DRIP-seq Optimized Peak Annotator), which makes use of gene expression data to annotate R-loop peaks to strand templates and expressed genes. Thus, DROPA allows the identification of the DNA strand annealed to the RNA and annotates R-loop.

## Implementation

### Program architecture and design

DROPA is a command-line tool, developed in Python, and it can be launched in Unix environment (Linux, MacOS, Windows Subsystem for Linux).

**Fig. 1:**
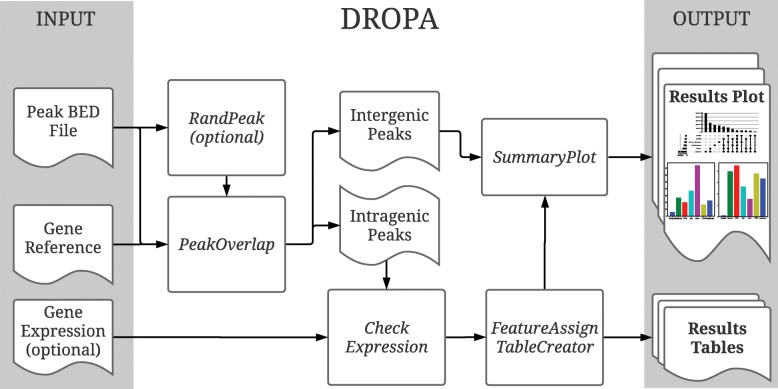
Overview of DROPA workflow

DROPA consists of six Python scripts, *PeakOverlap*, *CheckExpression*, *FeatureAssign*, *TableCreator*, *RandPeak*, and *SummaryPlot* (Fig. 1).

- *PeakOverlap* searches for genes overlapping R-loop peaks. Two BED (Browser Extensible Data) files are produced as output: one lists peaks with corresponding overlapping genes and the other lists intergenic peaks without overlapping genes. Notwithstanding there are some libraries in R (e.g. GenomicRanges [9]) that can perform this step, however we wrote *PeakOverlap* in Python to be consistent with the next scripts.
- *CheckExpression* introduces the main novelty of DROPA as compared with common peak annotation tools, as it considers gene expression levels in order to assign each R-loop peak to a given gene. It considers all overlapping genes of a peak and, if only one gene overlaps with the query peak, then that gene is assigned to the peak. In case of multiple genes overlapping to the same peak, *CheckExpression* evaluates their transcription levels and selects the gene with the highest level. Background levels can be set providing a threshold. If expression levels are below thresholds, they are the same for all overlapping genes, or if expression data are not provided, then the function selects the gene with the largest overlap with the query peak. Gene expression data can be in TPM (Transcripts Per Million), FPKM (Fragments Per Kilobase Million) or any other normalized values.
- *FeatureAssign* identifies all gene regions (upstream/downstream region, intron, exon, UTR (Untranslated Region) regions) overlapping to the peak.
- *TableCreator* returns a table that reports relevant information of annotated genes (name, template strand and other features) for each peak.
- *RandPeak* (optional) performs analyses of random R-loop peaks to calculate gene feature enrichment scores. The script takes the query peaks coordinates and returns randomly shuffled peaks all over the genome using BEDtools shuffle tool [10]. Then, it launches the 1 to 4 scripts of DROPA analyses for the random peaks.
- Once all the steps are performed, *SummaryPlot* is used to plot results.

### Input data

DROPA requires three input data, which are:

- A file containing query peak locations in BED format;
- A reference set folder containing information about genes features (5’UTR, 3’UTR, exon, intron) in BED format and a gene reference in BED12. We provide many ready-to-use reference set for *Homo sapiens* (hg19) and for *Mus musculus* (mm9 and mm10). Gene reference can be easily generated for every genome of interest, and also custom gene reference can be used.
- A 2-column gene expression table containing the name of each gene and its normalized expression value (FPKM, TPM, etc.). This table can be optional.

Besides the three input data, other custom parameters can be provided:

- The size of upstream and downstream regions.
- The gene expression threshold to consider a gene as expressed.
- The number of shuffle samples to perform the randomization analysis.

### Output data format

DROPA output consists of a folder containing table and image files.

The main table file result is the “*annotation.table*” that contains, for every annotated peak:

- Peak coordinates: chromosome, peak start, peak end;
- Peak name, as in the input file;
- Name of the gene, his strand and his expression value, as in the reference;
- Which features of the gene are covered by the peak (Upstream, 5’UTR, Exon, Intron, 3’UTR, Downstream).
- A warning flag if the peak is localized in a region in which antisense R-loops can form.

DROPA provides other secondary table files, such as the list of intergenic peaks and summary tables used to create plot figures. Figures and the annotation.table are provided in three version: the “expressed” in which are reported results for peaks annotated to genes with expression value above the threshold, the “unexpressed” in which are reported the ones annotated to genes with expression value below the threshold, and the “merged” which reports the aggregation of the previous two.

**Fig. 2:**
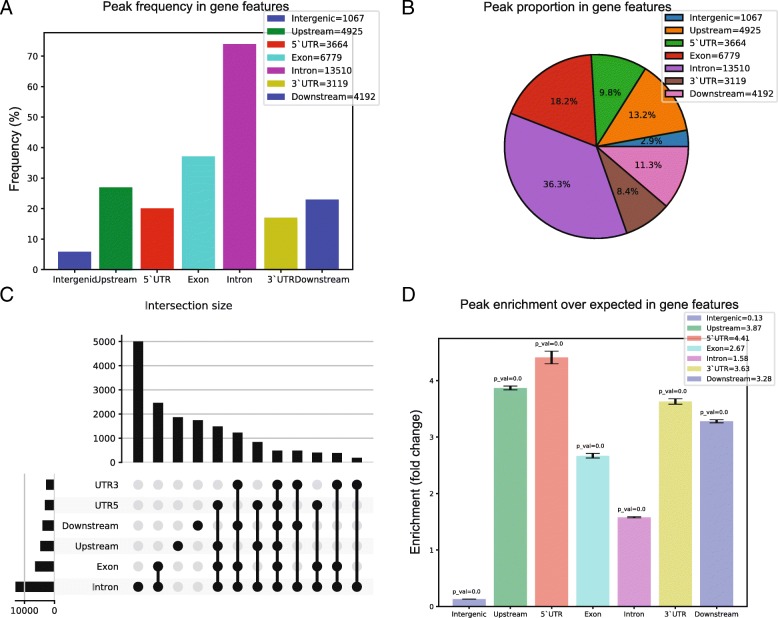
**a** Histogram showing the percentage (and the number in legend) of peaks that overlap each feature. **b** Pie chart showing the proportion of peak that overlap each feature (and the number in legend). **c** Upset plot showing how many peaks overlap more than one feature. **d** Histogram showing the fold enrichment between the number of peaks annotated to each feature and number of peaks shuffled over the genome

DROPA produces many informative summary plots, regarding the percentage of peaks overlapping each genic feature (Fig. 2a), or their proportion as a pie chart (Fig. 2b). Furthermore, since many peaks usually overlap more than one feature, DROPA provides a plot in which is shown the number of peaks that overlap each combination of feature (Fig. 2c). Finally, if enrichment analysis is performed, it is provided a histogram (Fig. 2d) with standard deviations bars and *p*-value of a chi-squared contingency test, showing the fold enrichment for each gene feature, calculated as the ratio between the number of query peaks that overlap a feature and the mean number of randomly shuffled peaks.

### Availability

DROPA package can be downloaded from https://github.com/marcrusso/DROPA.

### Installation

Detailed installation guide for DROPA and all python libraries required is available at https://github.com/marcrusso/DROPA. (see also Additional file 1 for DROPA requirements)

### Launching

To launch DROPA with default settings this command can be used:

python3 DROPA_v1.0.0.py -ref GeneReference/GeneReferenceSet/ -o OutputFolderName QueryPeak.bed

## Results

### Influence of expression data metrics on DROPA

As the main feature of DROPA is peak annotation using expression levels, we tested whether different expression metrics (TPM and FPKM) lead to different annotation output. In this analysis, the same default settings were used. The comparison showed that using TPMs more peaks (212, 1.3%) were assigned to expressed genes. However, all other peaks (15,872, 98.7%) were assigned to the same gene using FPKM or TPM values (see Additional file 1: Table S1). Overall the results show that using TPM or FPKM substantially leads to very similar overall peak annotation.

### Assessment of DROPA performance

To assess the correct annotation rate of DROPA, we determined the correct assignment of a query dataset of DRIPc-seq peaks [11] to the DNA template strand. DRIPc is a DRIP technique variant that maintains the strand information of DNA:RNA hybrid peaks. Our comparison shows that, when DROPA assigns peaks based on gene expression (76,526 peaks, see Additional file 1: Table S2), 88.6% (67,796) of them are assigned correctly (see Additional file 1: Table S3). Among the 11.4% (8730) of peaks with wrong annotation, we noticed that 5.05% (3871) are in the same position of another DRIPc peaks but in the opposite strand, and another 3.53% (2707) are mapped within 5000 bp upstream or downstream to expressed genes (see Additional file 1: Figure S1). As antisense transcription is known in particular at 5′ and 3′ ends of expressed genes, these analyses suggest that many peaks assigned to the wrong strand are potential antisense R-loops [11]. Therefore, if we consider only the transcribed regions of a gene, DROPA efficiency is 93.8% (see Additional file 1: Figure S1). In order to warn the user about peaks that can be ambiguously assigned as they map in regions where antisense R-loop can form, we provide: i) specific output tables of these peaks and ii) warning flags to these peaks in the main output tables, leaving therefore the user the option to exclude them for further analysis.

### DROPA comparison with existing tools

To assess DROPA performance, we compared it with three widely used or recent annotation tools: HOMER [12], PAVIS [13] and UROPA [14], which are based on different algorithms. PAVIS and HOMER annotate peaks based on the nearest TSS (Transcription Start Site), while UROPA allows to choose between the nearest start, end or center of the reference region. DROPA annotates all gene features (UTR regions, exons, introns, etc.) overlapping to peaks, while HOMER and PAVIS select one gene feature only. This may limit the biologically relevant information of HOMER and PAVIS output data when query peaks have a size larger than the gene features.

DROPA is highly flexible and customizable. DROPA, PAVIS and Homer have default gene reference sets that make these tools ready-to-use: DROPA has human (hg19) and mouse (mm9, mm10) genome as default, while PAVIS has gene sets of many organisms and genome assemblies. However, DROPA allows the choice of any custom gene set. Among the others, only PAVIS does not allow the use of a custom gene reference set. HOMER does not allow to set the size of upstream and downstream gene regions, which is useful while working on peaks that can form kilobases far from a gene, and it is instead customizable with DROPA, PAVIS and UROPA.

DROPA lacks a Graphical User Interface (GUI), however it is easy to use thanks to few and fully described command flags, which make it easily integrable into pipelines. PAVIS, a web-based tool, offers a GUI and requires an Internet connection, while UROPA offers both a command-line tool and a web-based GUI. HOMER is available only as command-line tool.

Although all four tools produce a full annotation table for every query peak, DROPA also produces summary tables and many other plots of peak distribution or enrichment over specific gene features. PAVIS produces a summary table and a pie chart of peak annotations, HOMER provides no plots but only a summary table, while UROPA also produces a summary report with plots.

Feature comparison summary is reported in Table 1.

**Table 1:** Feature comparison between DROPA and PAVIS, HOMER and UROPA

	DROPA	PAVIS	HOMER	UROPA
Offline Usage	✓	✗	✓	✓
Pipeline integration	✓	✗	✓	✓
Reference gene set customization	✓	✗	✓	✓
Upstream/downstream region definition	✓	✓	✗	✓
Multiple gene feature annotation	✓	✗	✗	✗
Statistical enrichment over gene feature	✓	✓	✓	✗
Summary plot Results	✓	✓	✗	✓

We compared the four tools output using an experimental set of R-loops peaks determined by DRIP-seq in human cells [6]. Peak calling was performed using MACS and comparisons were carried out using the same gene reference and the same upstream/downstream dimension. Briefly, DROPA gives different annotation results in comparison with all three tools in analysis. A full description of annotation result is reported in Additional file 1.

Intergenic peaks in DROPA data are always fewer compared to HOMER (10.8% of total query peaks versus 17.9% in HOMER), PAVIS (5.7% versus 12.1%) and UROPA (3.3% versus 11.2%). This is mainly due to the fact that DROPA does not take in account only the center of the query peak (that for peaks that have a dimension of kilobases can be far from the gene region) for annotation, but both the start and end point. About 10, 20 and 22% of query peaks are annotated with different genes by DROPA with respect to HOMER, PAVIS and UROPA, respectively.

As R-loop formation is mainly a co-transcriptional phenomenon and PAVIS and HOMER primarily rely on closest TSS, we can argue that DROPA identifies less false positive annotations as compared to PAVIS and HOMER due to the use of expression data (see Additional file 1: Figures S2 and S3). Although UROPA annotation does not rely on closest TSS search but rather on overlap, its annotation approach still gives a result clearly different from DROPA one (see Additional file 1: Figure S4), which, is optimized using gene expression data.

### Limitations

Even though DROPA can define and assign co-transcriptional peaks in the body of expressed genes with good efficiency, its main limitation is the detection and correct assignment of antisense R-loop peaks. To compensate for this limitation, DROPA provides a list of peaks assigned only to the upstream/downstream region of expressed gene, where antisense transcripts can be present. Moreover, in the main annotation table a warning flag indicates either peaks in upstream/downstream regions and peaks located at overlapping expressed genes. A comparison of this information with genomic datasets of antisense transcripts can help the user for further analysis.

## Conclusions

DROPA is a full customizable peak annotation tool optimized for co-transcriptional DRIP-seq peaks, allowing a finest gene annotation based on gene expression information. Since the expression data table is optional, this tool can be used with other sequencing data regarding genomic features that are not strictly associated with TSS (for which tools like PAVIS and HOMER are developed) and that are characterized by broad peak dimension, such as Histone marks IP-seq, DNAse-seq and FAIRE-seq. Using DROPA, users can take advantage from its alternative annotation algorithm, based on largest overlap with the query peak, the multi-feature annotation and the informative summary plots.

## Methods

In the following evaluation, DROPA was tested on a machine running Ubuntu OS (vers. 16.04 LTS) with 8 CPU cores and 16 GB of RAM.

### Influence of expression data metrics on DROPA

To perform evaluation of DROPA results using different gene expression values we used an experimental set of DRIP-seq peak (available at GEO: GSE115957) and his relative RNA-seq data. Using Stringtie (REF), TPM and FPKM were computed using RNA-seq data using RefSeq gene reference. Peak dataset and gene expression table are available in DROPA repository as Test_hg19_DRIP_peaks.bed. DROPA was launched two times with default settings using TPM or FPKM table as expression data and hg19_Refseq as gene reference. Results of annotation were compared counting how many peaks were annotated as intergenic and how many peaks were annotated to the same gene.

### Assessment of DROPA performance

To perform evaluation of DROPA using stranded data we downloaded a DRIPc-seq peak dataset and relative RNA-seq data (available at GEO: GSE70189). Peak dataset and gene expression table are available in DROPA repository. DROPA was launched with default settings using as input the peak dataset, the gene expression table and the gene reference hg19_UCSCgenes. Then strandness of peaks annotated on expressed genes was compared with the one of the original dataset.

### DROPA comparison with existing tools

In all 3 comparison we used an experimental set of DRIP-seq peak (available at GEO: GSE115957) and his relative RNA-seq data. Peak dataset and gene expression table are available in DROPA repository as Test_hg19_DRIP_peaks.bed and Test_hg19_RefSeq_Expression. Since each tool has different degree of customization (fixed upstream/downstream dimension, gene reference selection, etc.), we adapted DROPA settings to the one of the tool in analysis. In comparison with HOMER, DROPA was launched with upstream/downstream region dimensions set to 1 kb and RefSeq gene reference. HOMER was launched with default settings. In comparison with PAVIS, DROPA was launched with default settings and UCSCknown gene reference, while PAVIS was launched setting the Upstream/Downstream region to 5 kb (same as DROPA default). In comparison with UROPA, DROPA was launched with default settings and Ensembl gene reference, while UROPA was launched setting the Upstream/Downstream region to 5 kb. In all three comparison, after peak annotation, results were compared counting how many peaks were annotated as intergenic and how many peaks were annotated to the same gene.

### Availability and requirements

**Project name:** DROPA

**Project home page:** Source code on https://github.com/marcrusso/DROPA

**Operating system:** Unix (Linux or Mac OS or Windows Subsystem for Linux)

**Programming language:** Python3

**Other requirements:** All Python libraries requirements are listed in Supplementary. Bedtools software is required for peak randomization.

**License:** MIT license

**Any restrictions to use by non-academics:** None.

## Additional file

Additional file 1: Supplementary file containing DROPA requirements, summary tables and figures ragarding comparison results and a benchmark section. (DOCX 734 kb)

## Data Availability

DROPA repository containing source code, installation guide and usage information is available at https://github.com/marcrusso/DROPA. The datasets analyzed during the current study are available in the DROPA repository.

## Abbreviations

BED: Browser Extensible Data
ChIP-seq: Chromatin Immunoprecipitation
DRIP: DNA:RNA Immuno Precipitation
DROPA: DRIP Optimized Peak Annotator
FPKM: Fragments Per Kilobase Million
MACS: Model-based Analysis of ChIP-Seq
ssDNA: Single-strand DNA
TPM: Transcripts Per Million
TSS: Transcription Start Site
UTR: Untranslated Region
