# Supplementary material for: DROPA: DRIP-seq optimized peak annotator
Source: BMC Bioinformatics. 2019 Aug 6;20:414. doi: 10.1186/s12859-019-3009-9 (PMC6685255; doi:10.1186/s12859-019-3009-9)
Supplement: Supplementary file 1 — Supplementary file containing DROPA requirements, summary tables and figures ragarding comparison results and a benchmark section. (DOCX 734 kb) [file 12859_2019_3009_MOESM1_ESM.docx]

**Additional file 1**

**DROPA: DRIP-seq Optimized Peak Annotator**

**DROPA Requirements**

DROPA is developed and can be launched in UNIX environment from command-line. It uses Python3 (vers. Python 3.6) and requires five Python modules to run, which are listed below:

- numpy (vers. 1.16.1) [1]
- tqdm (vers. 4.31.1) (available at https://github.com/tqdm/tqdm/tree/v4.31.1)
- pandas (vers. 0.24.1) [2]
- intervaltree (vers. 3.0.2) (available at https://github.com/chaimleib/intervaltree)
- upsetplot (vers. 0.2.1)[3]
- matplotlib (vers. 3.0.3)[4]
- argparse (vers. 1.4.0)

For peak randomization bedtools (vers. 2.26.0) shuffle command is used.

**Influence of expression data metrics on DROPA**

In this evaluation we used the peak set Test_hg19_DRIP_peaks.bed and relative expression data computed as TPM and FPKM.

|  | DRIP-seq Query Peaks (18262 peaks) | |
| --- | --- | --- |
|  | FPKM | TPM |
| Peaks annotated on expressed genes | 15872 | 16084 |
| with same annotation | 15872 | 15872 |
| Peaks annotated (expressed+Unexpressed genes) | 16586 | 16586 |
| with same annotation | 16471 | 16471 |

**Table 1.** Summary of DROPA results using FPKM or TPM as expression data

Using TPM or FPKM for DROPA analysis does not affect annotation result. The only difference regards the presence of a higher number of peaks that are annotated to expressed genes. This is due to the fact that TPM values are higher than FPKM ones and since the same expression threshold is used, this results in an higher number of expressed genes using TPMs.

**Assessment of DROPA performance**

In this evaluation we used a DRIPc-seq peak dataset of 88310 peaks.

We launched DROPA with default settings and hg19_UCSC gene reference. The result of the annotation was:

| Intergenic peaks | 7,918 |
| --- | --- |
| Intragenic peaks on expressed genes (assigned with gene expression method) | 76,526 |
| Intragenic peaks on unexpressed genes (assigned with overlap method) | 3,866 |

**Table 2**. Summary of DROPA results using stranded DRIPc data.

Taking in account genes assigned on the base of gene expression:

| Intragenic peaks assigned with gene expression method | 76,526 |  |
| --- | --- | --- |
| Peaks assigned with correct strandness | 67,796 | 88.6% |
| Peaks assigned with wrong strandness | 8,730 | 11.4% |

**Table 3**. Summary of DROPA results using stranded DRIPc data for peaks annotated to expressed genes.

Regarding the 8,730 peaks assigned with wrong strandness, we noticed that they are particularly enriched in the upstream region outside the gene, contrary to the ones assigned with correct strandness, that are prevalently localized in the genebody (**Sup_FIG1**).

Moreover, of these 8,730 peaks, 3,871 are DRIPc peaks that are localized on the same region of another DRIPc peak, while 2,707 are associated with Upstream or Downstream region only.


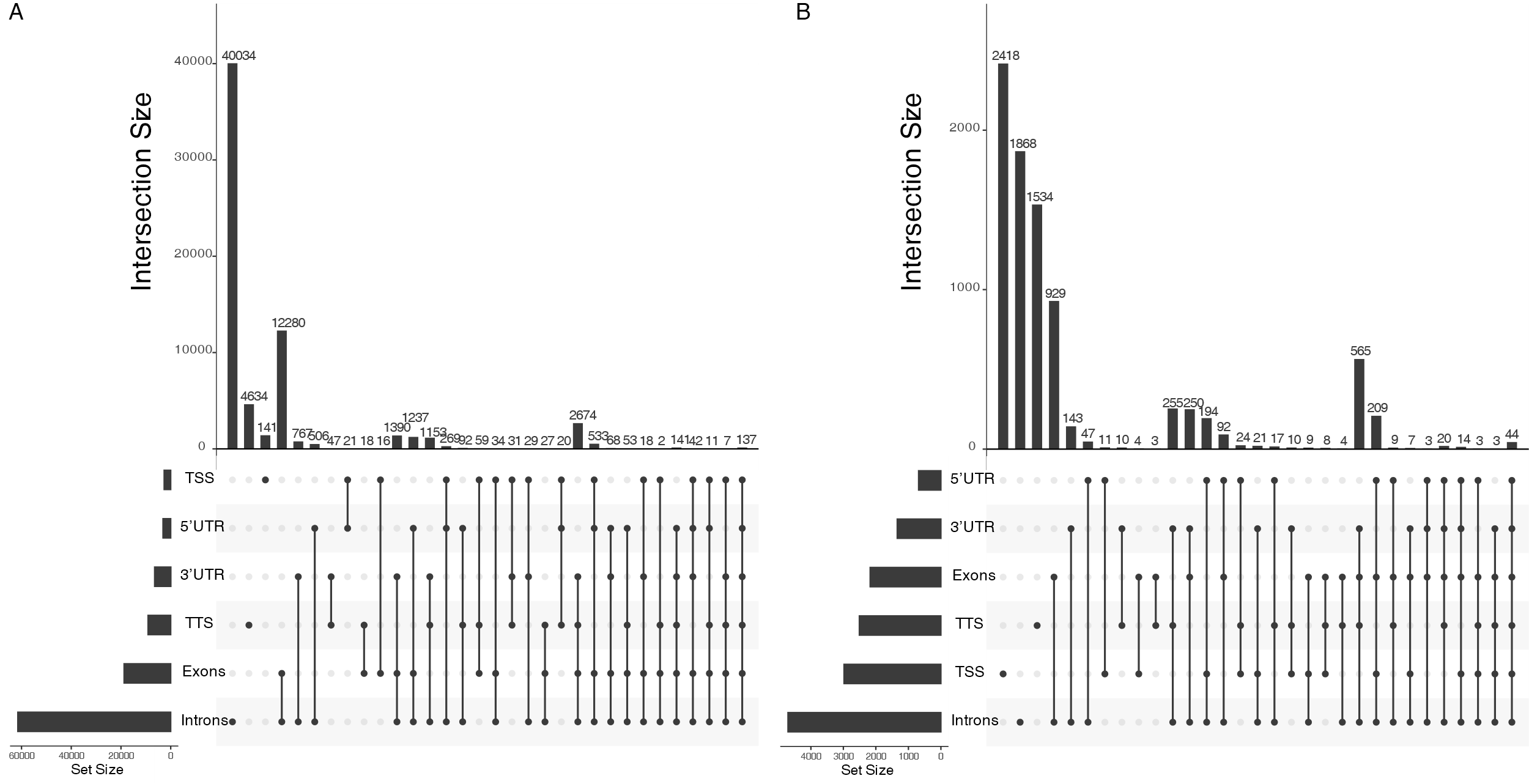


**Sup_FIG1**. On left: Upset plot showing the annotation of DRIPc peaks assigned to expressed genes with correct strandness. On right: Upset plot showing the annotation of DRIPc peaks assigned to expressed genes with wrong strandness.

**DROPA comparison with existing tools**

In the main text we described relevant features of DROPA compared to three annotation tools (HOMER, PAVIS and UROPA) and the results of a comparison of annotation output. Here we present a detailed description of the comparison. Since each tool has different degree of customization (fixed upstream/downstream dimension, gene reference selection, etc.), we adapted DROPA settings to the one of the tools in analysis.

**Comparison with HOMER**

HOMER tool provides some default dataset and allows to use custom datasets but it doesn’t allow to choose upstream/downstream region dimensions that are defined by default (1kb). In this comparison, we used the same reference gene set (RefSeq, the last version released in UCSC Table Browser) and the same upstream/downstream region dimensions (1kb).

All peaks that are annotated as intergenic in DROPA show the same annotation in HOMER which, however, show a higher number of intergenic peaks. This is due to HOMER algorithm: a) HOMER uses the center of the peak position and looks for the gene with the nearest TSS; b) it states the relative position of the center of the peak with respect to the gene; c) if the center of the peak is out of the gene region (plus 1kb upstream and downstream) it is annotated as intergenic.

Instead DROPA uses start and end coordinates and this allows a better intergenic/intragenic distinction. In Sup_Fig2A is shown an example of HOMER intergenic peaks that DROPA annotates as “Upstream”.

Regarding intragenic peaks, about 40% of query peaks are annotated to the same RefSeq ID, while 31,5% are annotated to different isoforms of the same gene (DROPA assign the peak to the most expressed one). About 10% of query peaks are annotated to different genes. Examples of different annotation are shown in Sup_Fig2B-C.

Therefore, DROPA seems to perform a better peak annotation than HOMER one, using start and end coordinates of the query peak, that is highlighted by smaller number of intergenic peaks. Furthermore, using a different approach of “peak to gene” annotation, we obtain a significative number of peaks annotated to different genes.

A summary of the comparison is shown in Table4.

|  | DRIP-seq Query Peaks (18262 peaks) | | |
| --- | --- | --- | --- |
|  | DROPA  Result | HOMER  Result | |
| Intergenic Peaks | 1978 | 3265 | |
| Intragenic Peaks | 16284 | 14997 | |
| Intragenic Peaks annotated to the same RefSeq ID |  | 7436 | |
| Intragenic Peaks annotated to different RefSeq ID |  | 7561 | |
|  |  | Same Gene Symbol | 5752 |
|  |  | Different Gene Symbol | 1809 |

**Table 4.** Summary of DROPA/HOMER comparison.

**Sup_Fig2.** A) Integrative Genome Viewer (IGV) screenshot of query peaks 100 and 101 that are annotated as intergenic in HOMER and respectively as 3’UTR/Downstream and Upstream/5’UTR to NM_18948 with DROPA. B) IGV screenshot of query peaks from 7542 to 7547. With HOMER, only
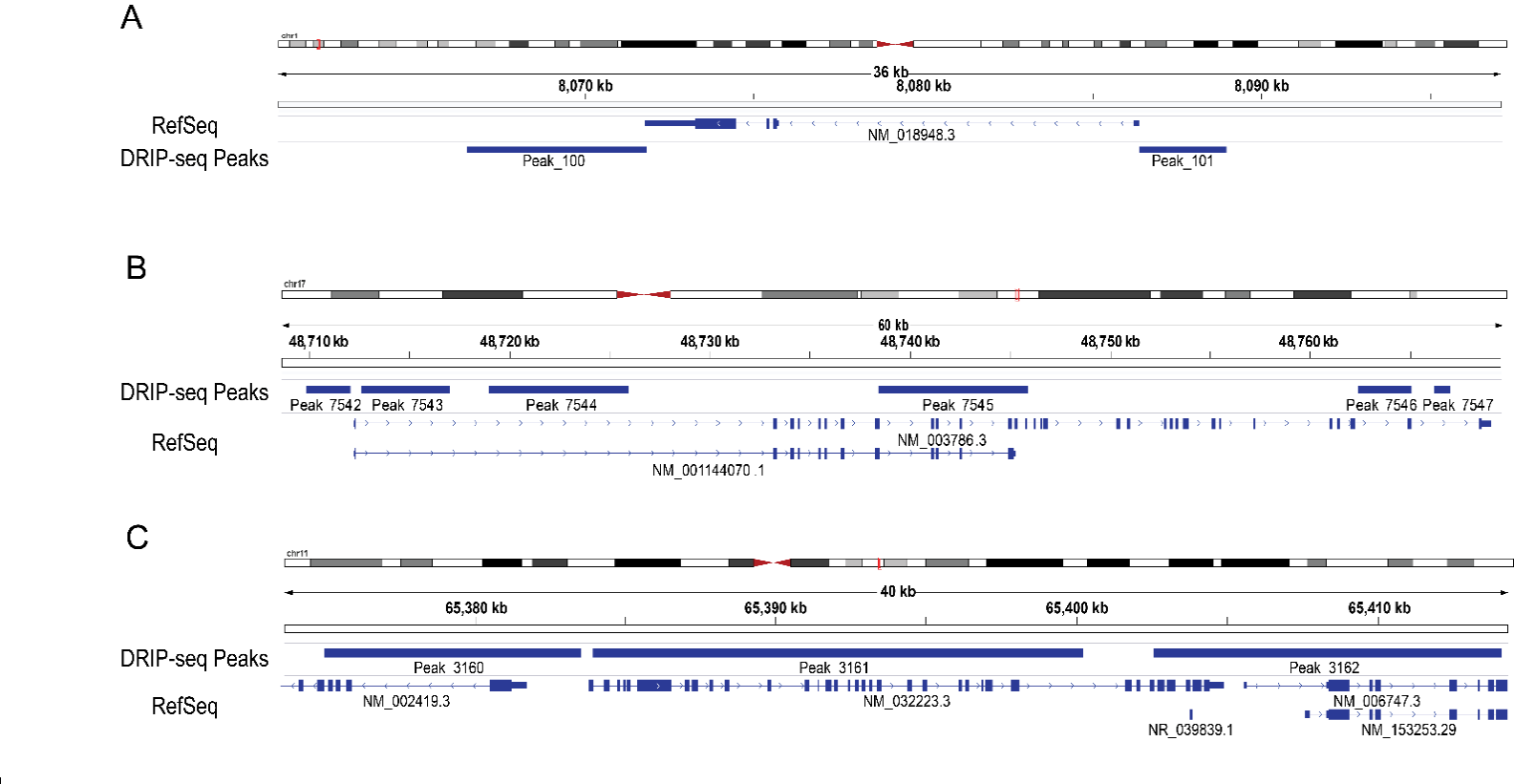
peaks 7543,7544 and 7545 are annotated to isoform NM_001144070, while peaks 7542, 7546 and 7547 are annotated as intergenic. Instead, DROPA annotates all these peaks to isoform NM_003786, which is the one with the higher expression level.

C) IGV screenshot of query peaks 3160,3161 and 3162. In this case HOMER annotate these peaks respectively to NM_002419, NM_032223 and NM_153253. Instead, DROPA annotates all them to the expressed gene, that is NM_032223.

**Comparison with PAVIS**

PAVIS provides reference gene set for many organisms and different version of genomes. For human genome version hg19 only UCSC “known genes” and “ref genes” are provided. It doesn’t allow to use a custom gene reference set.

In our comparison, we used the same upstream and downstream region dimension as for DROPA (5 kb) and the same reference gene set (UCSC known genes, the last version released on UCSC Table Browser).

Similarly to HOMER, PAVIS uses the approach of the gene with nearest TSS search.

Also in this case, all peaks that are annotated a intergenic in DROPA show the same annotation in PAVIS, that however shows an higher number of intergenic peaks. We noticed that peaks that PAVIS annotate as intergenic while DROPA doesn’t, are actually localized at gene level (Sup_Fig3A). This result could be explained by a lack of some gene coordinates in the reference set of PAVIS. But since it isn’t downloadable and since there are no further information about his version, we can’t be sure of that.

Regarding intragenic peaks, about 30% of query peaks are annotated to the same UCSC Gene ID, while 38.3% are annotated to different isoforms of the same gene (DROPA assign the peak to the most expressed one). About 20% of query peaks are annotated to different genes. Examples of different annotation are shown in Sup_Fig3B-C.

Also in this comparison, DROPA seems to perform a better annotation, obtaining a significantly different number of intergenic peaks and of peaks annotated to different genes. Anyhow, the impossibility to get the version of gene set used by PAVIS (that represent an important limitation) does not allow to know if these differences are mainly due to the gene set or to the different annotation method.

A summary of the comparison is shown in Table5.

|  | DRIP-seq Query Peaks (18262 peaks) | | |
| --- | --- | --- | --- |
|  | DROPA  Result | PAVIS  Result | |
| Intergenic Peaks | 1043 | 2212 | |
| Intragenic Peaks | 17219 | 16050 | |
| Intragenic Peaks annotated to the same UCSC Gene ID | - | 5510 | |
| Intragenic Peaks annotated to different UCSC Gene ID | - | 10540 | |
|  |  | Same Gene Symbol | 7002 |
|  |  | Different Gene Symbol | 3538 |

**Table 5.** Summary of DROPA/PAVIS comparison.


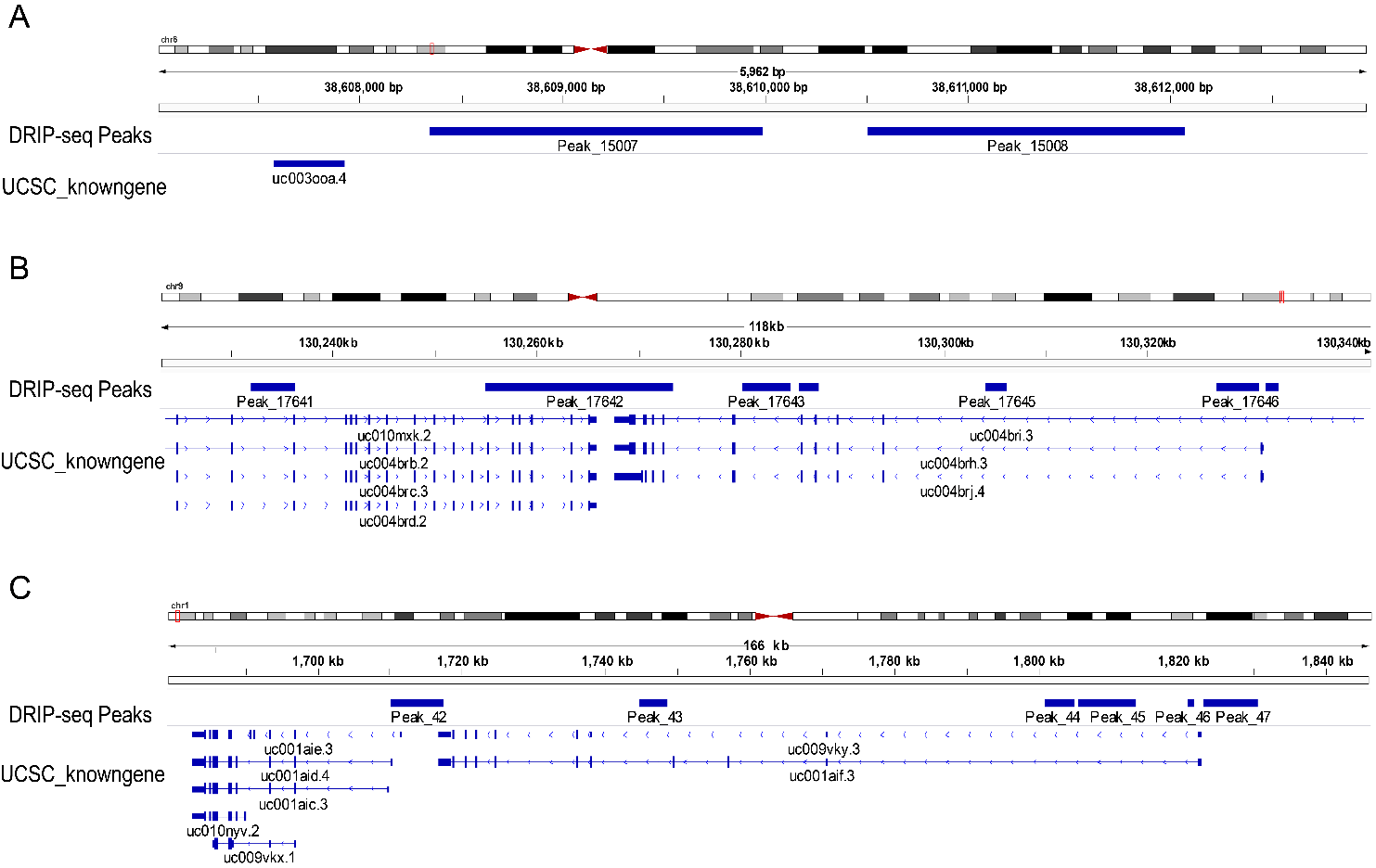


**Sup_Fig3.** A) IGV screenshot of query peaks 15007 and 15008 that are annotated as intergenic in PAVIS and respectively as Upstream to “uc003ooa” with DROPA. B) IGV screenshot of query peaks from 17641 to 17647. Peak 17641 is annotated to “uc004brb” with both DROPA and PAVIS. Peak 17642 is annotated to “uc004brb” by PAVIS, and to “uc004brj” (higher expression level) by DROPA. Peaks from 17643 to 17647 are annotated to “uc004bri” by PAVIS and to “uc004brj” (the expressed isoform) by DROPA. Interestingly, although peaks 17646 and 17647 are annotated to the same gene with both tools, with DROPA they are annotated to the TSS region of the gene. C) IGV screenshot of query peaks from 42 to 47. In this case, Peaks from 43 to 47 are annotated to “uc001aif” with both tools. Peak 42 is annotated to “uc001aie”, while with DROPA it is annotated to “uc001aif” too (due to the higher expression level).

**Comparison with** **UROPA**

UROPA can use any reference gene set provided by user and allows to set any upstream/downstream region. So, in our comparison, we set the upstream/downstream region of both DROPA and UROPA to 5000 and we used the same reference gene set (Ensembl). UROPA reports both a table with each gene the peak is annotated to, and a table with the best match. We used the latter one to compare his output to DROPA one.

Also in this case, all peaks that are annotated as intergenic in DROPA show the same annotation in UROPA, that however shows a higher number of intergenic peaks. In many cases UROPA annotates as intergenic peaks that actually overlaps a gene, as shown in Sup_Fig4.

Regarding intragenic peaks, only 16.71% of query peaks are annotated to the same Ensembl Transcript ID, while 49.7% are annotated to different isoforms of the same gene (DROPA assign the peak to the most expressed one). 22.3% of query peaks are annotated to different genes, and more than half of them (2689 peaks) are annotated on genes in opposite strand.

Also in this comparison, DROPA seems to perform a better annotation, obtaining a significantly different number of intergenic peaks and of peaks annotated to different genes.


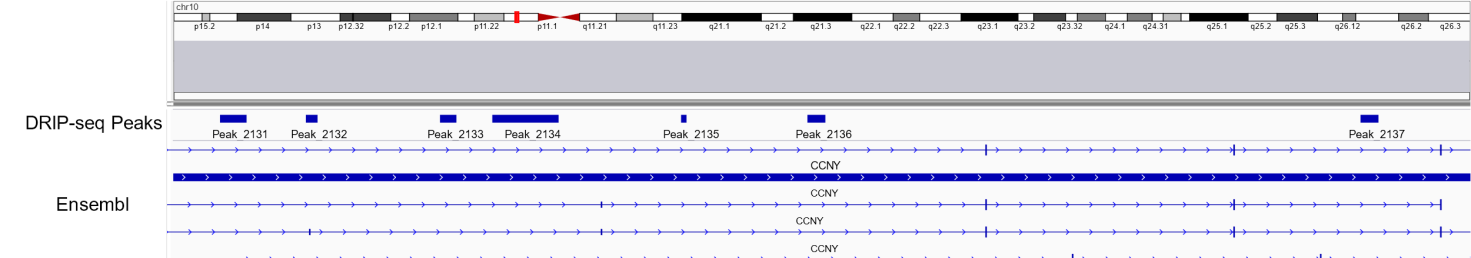
A summary of the comparison is shown in Table6.

**Sup_Fig4.** IGV screenshot of query peaks from 2131 to 2137. Peaks 2131, 2135 and 2137 are annotated as intergenic by UROPA, although they are clearly localized inside the gene. All the other peaks are annotated in the same way by DROPA and UROPA

|  | DRIP-seq Query Peaks (18262 peaks) | | |
| --- | --- | --- | --- |
|  | DROPA  Result | UROPA  Result | |
| Intergenic Peaks | 596 | 2049 | |
| Intragenic Peaks | 17666 | 16213 | |
| Intragenic Peaks annotated to the same Ensembl Transcript |  | 3052 | |
| Intragenic Peaks annotated to different Ensembl Transcript |  | 13161 | |
|  |  | Same Gene | 9077 |
|  |  | Different Gene | 4084 |

**Table 6.** Summary of DROPA/UROPA comparison.

**Benchmark**

We calculated the execution time for all tools but PAVIS (that is a web-tool), using the same peak dataset used the results comparison. Results are shown in Table 7.

|  | DROPA | DROPA  (with randomization set to 3) | HOMER | UROPA |
| --- | --- | --- | --- | --- |
| N. CPU used | 1 | 1 | 1 | 8 |
| Execution Time | 0m55,557s | 2m3,415s | 0m23,327s | 6m38,374s |

**Table 7**. Tools execution time comparison.

**Supplementary references**

1. Gold B, Cankovic M, Furtado L V., Meier F, Gocke CD. Do Circulating Tumor Cells, Exosomes, and Circulating Tumor Nucleic Acids Have Clinical Utility?: A Report of the Association for Molecular Pathology. J Mol Diagnostics. 2015;17:209–24. doi:10.1016/J.JMOLDX.2015.02.001.

2. McKinney W. Data Structures for Statistical Computing in Python. Proc 9th Python Sci Conf. 2010;1697900 Scipy:51–6. http://conference.scipy.org/proceedings/scipy2010/mckinney.html. Accessed 4 Jul 2018.

3. Lex A, Gehlenborg N, Strobelt H, Vuillemot R, Pfister H. UpSet: Visualization of Intersecting Sets. IEEE Trans Vis Comput Graph. 2014;20:1983–92. doi:10.1109/TVCG.2014.2346248.

4. Hunter JD. Matplotlib: A 2D Graphics Environment. Comput Sci Eng. 2007;9:90–5. doi:10.1109/MCSE.2007.55.
